# Methods for Specifying the Target Difference in a Randomised Controlled Trial: The Difference ELicitation in TriAls (DELTA) Systematic Review

**DOI:** 10.1371/journal.pmed.1001645

**Published:** 2014-05-13

**Authors:** Jenni Hislop, Temitope E. Adewuyi, Luke D. Vale, Kirsten Harrild, Cynthia Fraser, Tara Gurung, Douglas G. Altman, Andrew H. Briggs, Peter Fayers, Craig R. Ramsay, John D. Norrie, Ian M. Harvey, Brian Buckley, Jonathan A. Cook, for the DELTA group

**Affiliations:** 1 Institute of Health and Society, Newcastle University, Newcastle upon Tyne, United Kingdom; 2 Academic Urology Unit, University of Aberdeen, Aberdeen, United Kingdom; 3 Population Health, University of Aberdeen, Aberdeen, United Kingdom; 4 Health Services Research Unit, University of Aberdeen, Aberdeen, United Kingdom; 5 Warwick Evidence, University of Warwick, Coventry, United Kingdom; 6 Centre for Statistics in Medicine, Nuffield Department of Orthopaedics, Rheumatology and Musculoskeletal Sciences, University of Oxford, Oxford, United Kingdom; 7 Institute of Health and Wellbeing, University of Glasgow, Glasgow, United Kingdom; 8 Department of Cancer Research and Molecular Medicine, Norwegian University of Science and Technology, Trondheim, Norway; 9 Centre for Healthcare Randomised Trials, University of Aberdeen, Aberdeen, United Kingdom; 10 Faculty of Health, University of East Anglia, Norwich, United Kingdom; 11 National University of Ireland, Galway, Ireland; Institute of Psychiatry, King′s College London, United Kingdom

## Abstract

**Background:**

Randomised controlled trials (RCTs) are widely accepted as the preferred study design for evaluating healthcare interventions. When the sample size is determined, a (target) difference is typically specified that the RCT is designed to detect. This provides reassurance that the study will be informative, i.e., should such a difference exist, it is likely to be detected with the required statistical precision. The aim of this review was to identify potential methods for specifying the target difference in an RCT sample size calculation.

**Methods and Findings:**

A comprehensive systematic review of medical and non-medical literature was carried out for methods that could be used to specify the target difference for an RCT sample size calculation. The databases searched were MEDLINE, MEDLINE In-Process, EMBASE, the Cochrane Central Register of Controlled Trials, the Cochrane Methodology Register, PsycINFO, Science Citation Index, EconLit, the Education Resources Information Center (ERIC), and Scopus (for in-press publications); the search period was from 1966 or the earliest date covered, to between November 2010 and January 2011. Additionally, textbooks addressing the methodology of clinical trials and International Conference on Harmonisation of Technical Requirements for Registration of Pharmaceuticals for Human Use (ICH) tripartite guidelines for clinical trials were also consulted. A narrative synthesis of methods was produced. Studies that described a method that could be used for specifying an important and/or realistic difference were included. The search identified 11,485 potentially relevant articles from the databases searched. Of these, 1,434 were selected for full-text assessment, and a further nine were identified from other sources. Fifteen clinical trial textbooks and the ICH tripartite guidelines were also reviewed. In total, 777 studies were included, and within them, seven methods were identified—anchor, distribution, health economic, opinion-seeking, pilot study, review of the evidence base, and standardised effect size.

**Conclusions:**

A variety of methods are available that researchers can use for specifying the target difference in an RCT sample size calculation. Appropriate methods may vary depending on the aim (e.g., specifying an important difference versus a realistic difference), context (e.g., research question and availability of data), and underlying framework adopted (e.g., Bayesian versus conventional statistical approach). Guidance on the use of each method is given. No single method provides a perfect solution for all contexts.

*Please see later in the article for the Editors' Summary*

## Abstract 2

Jonathan Cook and colleagues systematically reviewed the literature for methods of determining the target difference for use in calculating the necessary sample size for clinical trials, and discuss which methods are best for various types of trials.

*Please see later in the article for the Editors' Summary*

## Editors' Summary

**Background:**

A clinical trial is a research study in which human volunteers are randomized to receive a given intervention or not, and outcomes are measured in both groups to determine the effect of the intervention. Randomized controlled trials (RCTs) are widely accepted as the preferred study design because by randomly assigning participants to groups, any differences between the two groups, other than the intervention under study, are due to chance. To conduct a RCT, investigators calculate how many patients they need to enroll to determine whether the intervention is effective. The number of patients they need to enroll depends on how effective the intervention is expected to be, or would need to be in order to be clinically important. The assumed difference between the two groups is the target difference. A larger target difference generally means that fewer patients need to be enrolled, relative to a smaller target difference. The target difference and number of patients enrolled contribute to the study's statistical precision, and the ability of the study to determine whether the intervention is effective. Selecting an appropriate target difference is important from both a scientific and ethical standpoint.

**Why Was This Study Done?:**

There are several ways to determine an appropriate target difference. The authors wanted to determine what methods for specifying the target difference are available and when they can be used.

**What Did the Researchers Do and Find?:**

To identify studies that used a method for determining an important and/or realistic difference, the investigators systematically surveyed the research literature. Two reviewers screened each of the abstracts chosen, and a third reviewer was consulted if necessary. The authors identified seven methods to determine target differences. They evaluated the studies to establish similarities and differences of each application. Points about the strengths and limitations of the method and how frequently the method was chosen were also noted.

**What Do these Findings Mean?:**

The study draws attention to an understudied but important part of designing a clinical trial. Enrolling the right number of patients is very important—too few patients and the study may not be able to answer the study question; too many and the study will be more expensive and more difficult to conduct, and will unnecessarily expose more patients to any study risks. The target difference may also be helpful in interpreting the results of the trial. The authors discuss the pros and cons of different ways to calculate target differences and which methods are best for which types of studies, to help inform researchers designing such studies.

**Additional Information:**

Please access these websites via the online version of this summary at http://dx.doi.org/10.1371/journal.pmed.1001645.

- Wikipedia has an entry on sample size determination that discusses the factors that influence sample size calculation, including the target difference and the statistical power of a study (statistical power is the ability of a study to find a difference between treatments when a true difference exists). (Note: Wikipedia is a free online encyclopedia that anyone can edit; available in several languages.)
- The University of Ottawa has an article that explains how different factors influence the power of a study

## Introduction

A randomised controlled trial (RCT) is widely regarded as the preferred study design for comparing the effectiveness of health interventions [1]. Central to the design and validity of an RCT is a calculation of the number of participants needed: the sample size. This provides reassurance that the study will be informative. Using the Neyman-Pearson method (a conventional approach to sample size calculation), a (target) difference that the RCT is designed to detect is typically specified.

Selecting an appropriate target difference is critical. If too small a target difference is estimated, the trial may be a wasteful and an unethical use of data and resources. If too large a target difference is hypothesized, there is a risk that a clinically relevant difference will be overlooked because the study is too small. Both extremes could therefore have a detrimental impact on decision-making [2]. Additionally, through its impact on sample size, the choice of target difference has substantial implications in terms of study conduct and associated cost.

However, unlike the statistical considerations involved in sample size calculation, research on how to specify the target difference has been greatly neglected, with no substantive guidance available [3],[4]. While a variety of potential approaches have been proposed, such as specifying what an important difference would be (e.g., the “minimal clinically important difference”) or what a realistic difference would be given the results of previous studies, the current state of the evidence base is unclear. Although some reviews of different types of methods have been conducted [2],[5], there is still a need for a comprehensive review of available methods. The aim of this systematic review was to identify potential methods for specifying the target difference in an RCT sample size calculation, whether addressing an important difference (a difference viewed as important by a relevant stakeholder group [e.g., clinicians]) and/or realistic difference (a difference that can be considered to be realistic given the interventions to be evaluated). The methods are described, and guidance offered on their use.

## Methods

A comprehensive search of both biomedical and selected non-biomedical databases was undertaken. Search strategies and databases searched were informed by preliminary scoping work. The final databases searched were MEDLINE, MEDLINE In-Process, EMBASE, the Cochrane Central Register of Controlled Trials, the Cochrane Methodology Register, PsycINFO, Science Citation Index, EconLit, Education Resources Information Center (ERIC), and Scopus (for in-press publications) from 1966 or earliest date coverage; the searches were undertaken between November 2010 and January 2011. Given the magnitude of the literature identified by this initial search and the belief that updating the search would not lead to additional approaches of specifying the target difference, an update of this search was not carried out. There was no language restriction. It was anticipated that reporting of methods in the titles and abstracts would be of variable quality and that therefore a reliance on indexing and text word searching would be inadvisable. Consequently, several other methods were used to complement the electronic searching and included checking of reference lists, citation searching for key articles using Scopus and Web of Science, and contacting experts in the field. The protocol and details of the search strategies used are available in Protocol S1 and Search Strategy S1.

Additionally, textbooks covering methodological aspects of clinical trials were consulted. These textbooks were identified by searching the integrated catalogue of the British Library and the catalogues (for the most recent 5 y) of several prominent publishers of statistical texts. The project steering group was also asked to suggest key clinical trial textbooks that could be assessed. Because of the nature of the review, ethical approval was unnecessary.

To be included in this review, each study had to report a formal method that had been used or could be used to specify a target difference. Any study design for original research was eligible, provided its assessment was based on at least one outcome of relevance to a clinical trial. Studies were excluded only if they were reviews, failed to report a method for specifying a target difference, reported only on statistical sample size considerations rather than clinical relevance, or assessed an outcome measure (e.g., number needed to treat) without reference to how a difference could be determined.

Potentially relevant titles and abstracts were screened by either or both of two reviewers (J. H. or T. G.), with any uncertainties or disagreements discussed with a third party (J. A. C.). Full-text articles were obtained for the titles and abstracts identified as potentially relevant. These were provisionally categorised according to method of specifying the target difference (if detailed in the abstract). One of four reviewers (J. H., T. G., K. H., or T. E. A.) screened the full-text articles and extracted information, after having screened and extracted information from a practice sample of articles and compared results to ensure consistency in the screening process. Where there was uncertainty regarding whether or not a study should be included for data extraction, the opinion of a third party (J. A. C.) was sought, and the study discussed until consensus was reached.

Data were extracted on the methodological details and any noteworthy features such as unique variations not found in other studies reporting the same method. Specific information relevant to each particular method was recorded, and no generic data extraction form was used across all methods. It was felt that a generic data extraction form that included all fields of relevance to all methods would be too cumbersome, because the methods varied in conception and implementation.

Narrative descriptions of each method were produced, summarising the key characteristics based on extracted data on the similarities and differences in each application of the same method, frequency with which each variant of the method was used, and strengths and weaknesses of the method, either identified by the review team as potentially important, or extracted from study authors' own points about the strengths and limitations of their method (or methods) as reported in the articles. Methods were assessed according to criteria developed by the steering group prior to undertaking the evidence synthesis; the criteria covered the validity, implementation, statistical properties, and applicability of each method. The initial assessment was carried out by J. A. C. and revised by the steering group.

## Results

**Figure 1:**
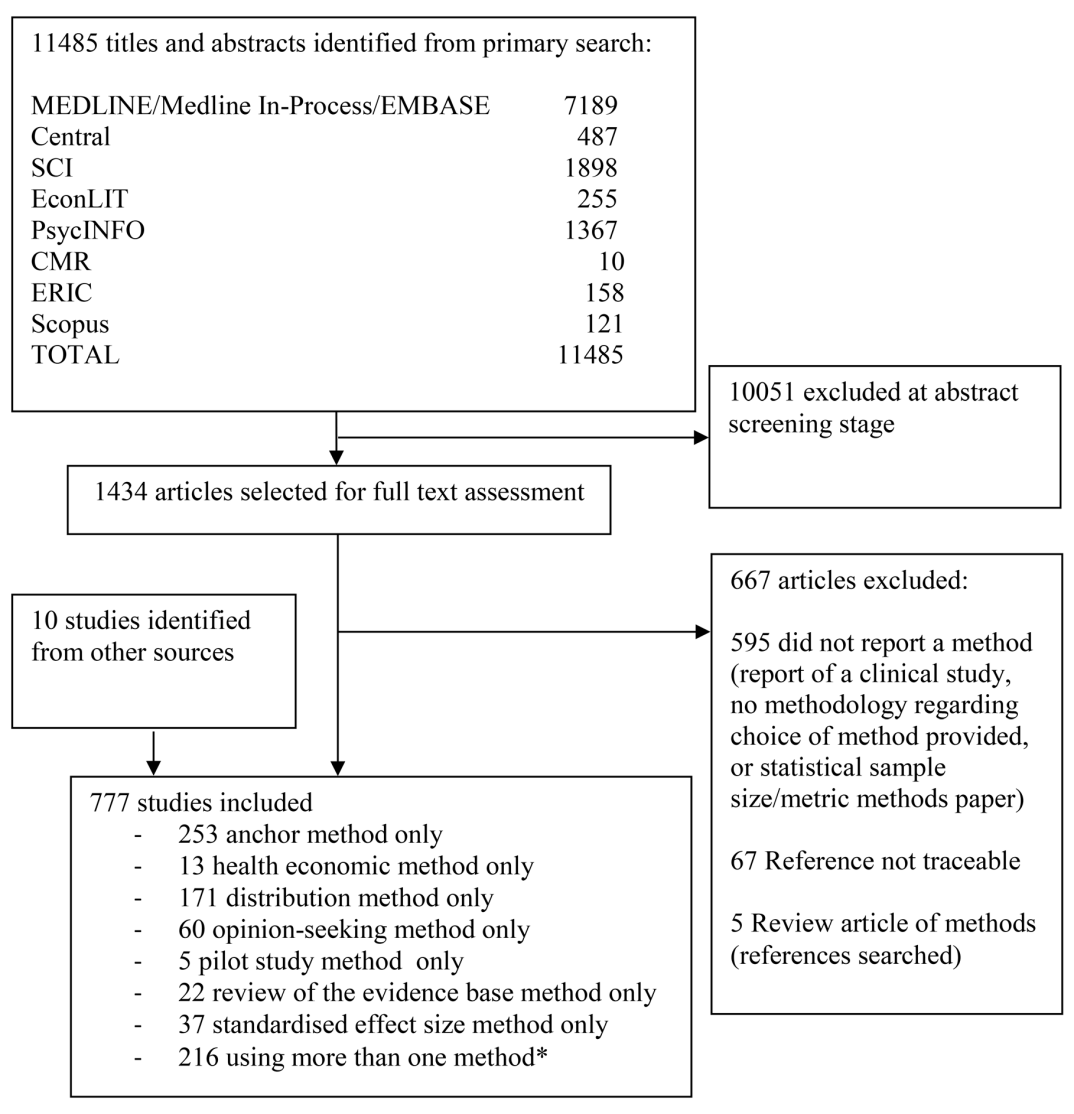
PRISMA flow diagram. *For a breakdown of studies that used more than one method in combination, please see Table 1. Central, Cochrane Central Register of Controlled Trials; CMR, Cochrane Methodology Register; ERIC, Education Resources Information Center; SCI, Science Citation Index.

We identified 11,485 potentially relevant studies from the databases searched. The number of studies found within each database is detailed in Figure 1 (PRISMA flow diagram), showing the number of studies for each method.

Of the potentially relevant studies identified, 1,434 were selected for full-text assessment; a further nine were identified from other sources. Fifteen clinical trial textbooks and the International Conference on Harmonisation of Technical Requirements for Registration of Pharmaceuticals for Human Use tripartite guidelines were also reviewed, though none identified a method that had not already been identified from the journal database searches. In total, 777 studies were included. Seven methods were identified—anchor, distribution, health economic, opinion-seeking, pilot study, review of the evidence base, and standardised effect size (SES). Descriptions of these methods are provided in Box 1. No methods were identified by this review beyond those already known to the reviewers. The anchor, distribution, opinion-seeking, review of the evidence base, and SES methods were used in studies in varied clinical and treatment areas, but predominantly in those pertaining to chronic diseases. Although the number of included studies for both the health economic and pilot study methods was much smaller, real or hypothetical trial examples covered pharmacological and non-pharmacological treatments for both acute and chronic conditions.

**Box 1. Methods for Specifying an Important and/or Realistic Difference**

**Methods for specifying an important difference**

- **Anchor:** The outcome of interest can be “anchored” by using either a patient's or health professional's judgement to define an important difference. This may be achieved by comparing a patient's health before and after treatment and then linking this change to participants judged to have shown improvement/deterioration. Alternatively, a more familiar outcome, for which patients or health professionals more readily agree on what amount of change constitutes an important difference, can be used. Alternatively, a contrast between patients can be made to determine a meaningful difference.
- **Distribution:** Approaches that determine a value based upon distributional variation. A common approach is to use a value that is larger than the inherent imprecision in the measurement and therefore likely to represent a minimal level for a meaningful difference.
- **Health economic:** Approaches that use principles of economic evaluation. These typically include both resource cost and health outcomes, and define a threshold value for the cost of a unit of health effect that a decision-maker is willing to pay, to estimate the overall net benefit of treatment. The net benefit can be analysed in a frequentist framework or take the form of a (typically Bayesian) decision-theoretic value of information analysis.
- **Standardised effect size:** The magnitude of the effect on a standardised scale defines the value of the difference. For a continuous outcome, the standardised difference (most commonly expressed as Cohen's d “effect size”) can be used. Cohen's cutoffs of 0.2, 0.5, and 0.8 for small, medium, and large effects, respectively, are often used. Thus a “medium” effect corresponds simply to a change in the outcome of 0.5 SDs. Binary or survival (time-to-event) outcome metrics (e.g., an odds, risk, or hazard ratio) can be utilised in a similar manner, though no widely recognised cutoffs exist. Cohen's cutoffs approximate odds ratios of 1.44, 2.48, and 4.27, respectively. Corresponding risk ratio values vary according to the control group event proportion.

**Methods for specifying a realistic difference**

- **Pilot study:** A pilot (or preliminary) study may be carried out where there is little evidence, or even experience, to guide expectations and determine an appropriate target difference for the trial. In a similar manner, a Phase 2 study could be used to inform a Phase 3 study.

**Methods for specifying an important and/or a realistic difference**

- **Opinion-seeking:** The target difference can be based on opinions elicited from health professionals, patients, or others. Possible approaches include forming a panel of experts, surveying the membership of a professional or patient body, or interviewing individuals. This elicitation process can be explicitly framed within a trial context.
- **Review of evidence base:** The target difference can be derived using current evidence on the research question. Ideally, this would be from a systematic review or meta-analysis of RCTs. In the absence of randomised evidence, evidence from observational studies could be used in a similar manner. An alternative approach is to undertake a review of studies in which an important difference was determined.

Substantial variation between studies was found in the way the seven methods were implemented. In addition, some studies used several methods, although the combinations used varied, as did the extent to which results were triangulated. The anchor method was the most popular, used by 447 studies, of which 194 (43%) used it in combination with another method. The distribution method was used by 324 studies, of which 153 (47%) used it alongside another method. Eighty studies used an opinion-seeking method, of which 20 (25%) also used additional methods. Twenty-seven studies used a review of the evidence base method, of which five (19%) also used another method. Six studies used a pilot study method, of which one (17%) also used another method. The SES method was used by 166 studies, of which 129 (78%) also used another method. Thirteen studies used a health economic method.

For all methods used in combination with others, Table 1 provides a breakdown of the variety of combinations identified and their frequency. The main variations identified from the systematic review for each of the methods are described in Table 2, and are further described in the text below. A brief summary of the literature for each method is given below and also of studies that used a combination of methods. Table 3 contains an assessment of the value of the individual methods. Table 4 contains examples and key implementation points for the use of each method.

**Table 1:** Use of multiple methods.

Methods Used in Combination							Number of Studies
Anchor	Distribution	Health Economic	Opinion-Seeking	Pilot Study	Review of Evidence Base	Standardised Effect Size	
√	√						70
√	√					√	63
√						√	46
	√					√	13
√			√				8
	√		√				3
√	√		√				2
√			√			√	2
			√		√		2
√					√		1
√	√		√			√	1
√			√		√	√	1
	√		√			√	1
			√			√	1
					√	√	1
				√	√		1

**Table 2:** Main variations in implementation of the methods.

Anchor	Distribution	Health Economic	Opinion-Seeking	Pilot Study	Review of the Evidence Base	Standardised Effect Size
**Two main areas of variation:** **1. Anchor design**•Judgement based anchor (e.g., patient's, health professional's, or carer's); judgements can be changes in individual over time or contrasting between individuals•The number of points on the anchor instrument (Likert scale, VAS) [8],[9],[12]•Objective measurements (e.g., ≥5 mm toenail growth) as the anchor [26]•Using a measure with an accepted definition of importance as an the anchor**2. Determination of important difference**•Considering deterioration as well as improvement [7],[18],[22]•Calibrating for no change group for within-person anchoring [7],[18],[22]•Utilising receiver operating characteristic curve approach to trade off probability of failure to detect an important difference versus falsely concluding an important difference when there is none [11]	**Three main approaches:** **1. Measurement-error-based approach**•Calculation of the SEM, typically defined as 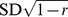 , where *r* is a measure of reliability such as Cronbach's alpha [2],[42]–[44],[49]–[51]. Various multiplicative factors and definitions of the SEM have been proposed [2]. The SEM is typically based upon the maximum error associated with two repeat within-person measurements.•Jacobson and colleagues proposed two similar approaches [39],[47],[48],[53]: (i) the RCI, which incorporates the SEM and a confidence level for the estimate; the mean change in scores is divided by 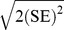 , where 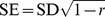 , with an RCI above 1.96 typically used as a cutoff; variants of this formula exist [2]; and (ii) beyond a plausible (95%) limit of agreement, e.g., 2SD of the mean score; a “normative” reference population can also be used in both approaches**2. Statistical-test-based approach**Smallest difference that could be statistically detected [56]; variants exist depending on data collected and planned statistical analysis, e.g., two independent groups (equal size and variance) [41],[46] **3. Rule-of-thumb-based approach**Defines an important difference based upon the distribution of the outcome, i.e., using a substantial fraction of the possible range; for example, using 10 mm on a 100-mm VAS measuring symptom severity [54] or a proportion of all the possible response level changes that could possibly be achieved [38]	**Four main approaches:** **1. Incremental cost per unit approach**Identifying the difference in effectiveness that leads to the incremental cost per unit of health being less than/equal to a decision-maker's WTP threshold [58],[59] or to equivalence between trial interventions [63]; the cost of the study and avoiding disabilities can be considered [64],[65] **2. Net benefit**WTP multiplied by the difference in effectiveness minus the difference in costs between interventions [62] **3. Maximising “cost efficiency”**The ratio of expected scientific/clinical/practical value for a given sample size, over the cost of conducting a study of that sample size [57] **4. Optimal sample size approach**Calculation based on perspective of profit maximisation (where expected net gain is a profit function) or single payer system (where the objective is to maximise net benefit) [60],[61],[122]	**Four main areas of variation:**1. Whose opinion is being sought (clinicians, patients, trialists) [66],[67],[69],[70],[72],[74],[75],[77]–[79],[81]–[83]2. Method used to elicit opinions (interviews, surveys, or both; frequency of data collection) [76]3. Complexity of the data elicited, e.g., asking for a value considered to be clinically significant, ranking criteria in terms of their importance, preference regarding hypothetical scenarios up to full (Bayesian) specification of distribution [67],[68],[75]4. Approach adopted to consolidate multiple responses: use a simple numerical summary (e.g., mean) [76],[80], Delphi method [84]–[86], or a proportion, e.g., “the majority” (i.e., >50%) [86]	**Two approaches to using observed values**1. Fully specify the target difference (e.g., mean difference and SD)2. Partly specify the target difference (e.g., using the observed SD or control proportion only) [88]; substantial uncertainty will still typically exist, though adjustment for this can be made [89]	**Three main areas of variation:**1. Reviewing previous studies to determine an important and/or realistic difference to specify the target difference [94],[99],[102],[103]2. Approach adopted to combine/choose between study results, e.g., using meta-analysis summary to determine a conclusive value [96]–[98]; alternatively, using observed values to fully or partly specify the target difference, e.g., mean difference and SD or coefficient of variation (equivalence trial) [99],[102],[103]3. Going beyond current literature by conducting a simulation study of the impact of adding a new study into a meta-analysis of studies (allowing for current uncertainty) to determine the size of a new trial given the required statistical power and significance level [101]	**Two main areas of variation:** **1. Values used for SES formula (Cohen's d)**•Mean used, SD from comparing between groups, or within one group (before and after) used; baseline SD or change score or pooled SD of two time points (baseline and follow-up) [104],[115] or the largest SD value [112]•Comparison of data with a reference population that serves as normative data [110],[114] **2. Alternative formulas**•Examples include a “modified Cohen's d” with correction for SD of change scores to account for within-person correlation [113] or correcting resulting effect size for this [104];Dunlap's d formula to compare effect sizes between treatment and placebo groups at allow for multiple follow-up measurements [107]

RCI, reliable change index; VAS, visual analogue scale; WTP, willingness to pay per unit of effectiveness.

**Table 3:** Assessment of the value of the methods.

Criteria	Method						
	Anchor	Distribution	Health Economic	Opinion-Seeking	Pilot Study	Review of the Evidence Base	Standardised Effect Size
**Validity**							
Does the method seem a sensible approach)? (face validity)	Yes	No	Yes	Yes	Yes	Yes	Yes
Does the method allow the overall benefit/harm profile of a treatment comparison to be addressed? (content validity)	As it is based upon a single outcome, the scope is limited; multiple perspectives can be accommodated	Focuses upon a single outcome and does not address directly either a realistic or an important difference	Potentially the most comprehensive approach, though it can be complex, data-hungry, and time-intensive; a value judgement is needed as to whose costs and benefits are important	Yes, though conditional upon a perspective	Yes	Yes	No
Has the method been shown to be consistent with an independent standard? (criterion validity)	Yes	No	No, usage so far has been in hypothetical retrospective examples	No	No	No	No, with an exception for some quality of life outcomes
Has the method been shown to be consistent with expected drivers (e.g., is the specified difference greater when there is a larger risk of harm)? (construct validity)	Yes	Findings have been conflicting	No, usage so far has been in hypothetical retrospective examples	No	Yes	Yes	No
**Implementation**							
Has the method been reported clearly enough to be reproducible (i.e., reviewers can easily agree upon reading what the method was and how it was applied)?	Yes	Yes	Yes, although the complexity of some of the approaches may require extensive reporting	Yes	Yes	Yes	Yes
Are there any important variations in implementation?	Yes	Yes	Yes	Yes	Yes	Yes	Yes
**Statistical properties**							
Has the method's repeatability been assessed (consistency of estimate when repeated—if applicable)?	Yes	Yes	No, although in principle for a given model structure and data inputs, the approach is repeatable	No	No	Yes	Not applicable
Is uncertainty of the estimated difference addressed by the method (implicitly or explicitly)?	Yes	Yes	Yes, using the more complex approaches	Yes, when adopting a synthesis of opinion	Yes	Yes, where the result from an appropriate statistical analysis is used	No
Has the method been shown to be sensitive to different outcomes/populations?	Yes	Yes	No	Yes, to a limited extent	Yes	Yes	No; universal values are routinely applied irrespective of the outcome and population
**Applicability**							
Is the method suited to any trial design?	Yes	Yes	Yes	Yes	Yes, though it is more likely to be used for Phase 3 or definitive trials	Yes, though it is more likely to be used for Phase 3 or definitive trials	Yes
Can the method be used for a variety of outcome measures?	Continuous/ordinal outcome only	Continuous/ordinal outcome only	Yes	Yes	Yes	Yes	Yes, though it is widely used only for a continuous outcomes
Is the method acceptable to patients, clinicians, and trialists?	Yes	Uncertain	Uncertain	Yes	Yes	Yes	Uncertain, though widely used
Is it straightforward to use?	Yes	Yes	No, except for simpler, more naive approaches	Yes	Yes, though it requires a study to be carried out	Yes, though it requires a review to be carried out	Yes
Has the method been used in an RCT setting?	Yes	Yes	Published examples are retrospective	Yes	Yes	Yes	Yes

**Table 4:** Usage of methods—examples and key implementation points.

Method	Example	Key Points
**Anchor**	Neuropathy Total Symptom Score-6 was measured at baseline and 1 y in patients with diabetes mellitus and diabetic peripheral neuropathy. The clinical global impression anchor—a seven-point scale ranging from marked improvement to marked worsening, which assesses the change in health status between baseline and 1 y—was collected by a health professional [8].	• Suitable for continuous (or ordinal) outcomes.• Anchor implementation is critical, e.g., the perspective and anchor adopted.• Particularly suited to quality of life measures.• The magnitude of the difference can be sensitive to the population group (e.g., ceiling/floor and disease severity effects may exist).• Use of the most common anchor approach implies that a within-person (important) difference can be applied, though a between-person approach is also possible.
**Distribution**	The Norwegian Fear Avoidance Beliefs Questionnaire (FABQ) was completed by 28 patients with chronic lower back pain. Using a measurement error approach, the maximum difference that could be attributed to spurious variation for the FABQ-Work and FABQ-Physical Activity scales was calculated as 12 and 9 units, respectively. These values can be considered as a lower bound of an important difference for the corresponding scale and can be used with an appropriate SD value [45].	• Suitable for continuous (or possibly ordinal) outcomes.• Use of the distribution method (i.e., measurement error approach) is of limited merit because of its weak justification of an “important” difference.• A simple range or levels approach should be a last resort if no more informative methods can be used, and only when the outcome has clear meaning.
**Health economic**	For women with tubal damage, IVF or tubal surgery could be used to treat infertility. The cost per pregnancy was calculated for both treatments. Based upon existing data, surgical treatment is successful in 12% of cases. Given this estimate, the required proportion of successful treatments for the more expensive IVF treatment was calculated as 27%, and a difference of 15% (27% to 12%) was considered (economically) important [64].	• Allows a comprehensive approach to the value of an RCT; in particular, the costs of the intervention and its comparator and of research can be considered in conjunction with possible benefits and consequences of decision-making. The flexible modelling framework allows any type of outcome to be incorporated.• The perspective adopted is critical—the viewpoint and values that are used to determine the scope of costs and benefits incorporated into the model structure.• Uncertainty around inputs can be substantial, and extensive sensitivity analyses will likely be needed. Some inputs (e.g., time horizon) will be particularly challenging to specify, as well as appropriately representing the statistical relationship of multiple parameters. These could also be based on empirical data and/or expert opinion.• This can be a resource-intensive and complex approach to determining the sample size.• Unlikely to be accepted as the sole basis for study design at present despite intuitive appeal. Patients and clinicians may be resistant to the formal inclusion of cost into the design and thereby the primary interpretation of studies. Expressing the difference in a conventional way is likely to be necessary, as it is more intuitive to stakeholders and also furthers the science of interventions. It could provide additional justification for conducting a large and expensive trial (e.g., when there is a small effect and/or events are rare).
**Opinion-seeking**	Six experts were asked to recommend an important difference for the Doyle Index to be used in a hypothetical trial of two antirheumatic drugs with stated inclusion/exclusion criteria for patients with rheumatoid arthritis. A Delphi consensus-reaching approach with three rounds was implemented by mail. The median (range) estimate for the third round was 5.5 (5.7), and 5.5 could be viewed as an important difference and used with an appropriate SD value [71].	• Allows for varying degrees of complexity of the scenario (e.g., consideration of related effects or impact on practice) and any outcome type (binary, continuous, or survival).• The perspective is critical—whose opinions are being sought.• A realistic and/or important target difference can be sought.• A target difference that takes into account other outcomes and/or consequences (e.g., a target difference that would lead to a health professional changing practice) or focuses exclusively on a single outcome can be sought.
**Pilot study**	A pilot trial compared a cognitive behavioural therapy to physiotherapy in patients with acute lower back pain. The SD of Roland–Morris scores was calculated as 5.7, which was used in combination with an estimate of an important difference of 4 from a previous study [87].	• There is a need to assess the relevance of the pilot study to the design of a new RCT study. Some down-weighting (whether formally or informally) may be needed according to the relevance of the study and methodology used. For example, a Phase 2 study should be used to directly specify a (realistic) target difference for a Phase 3 study only if the population and outcome measurement are judged to be sufficiently similar.• Helpful for estimating outcome components such as variability of a continuous outcome (or control group rate for a binary outcome), although the estimation of the target difference is typically imprecise because of a small sample size.• This approach can be used in conjunction with another method (e.g., using an opinion-seeking method to determine an important difference) to allow full specification of the target difference.
**Review of the evidence base**	A systematic search of an online medical database identified no RCTs that had compared acupuncture to a waiting list control for patients with breast cancer and assessed fatigue. Two further searches identified relevant studies from which an estimate of the within-group effects upon fatigue for acupuncture and waiting list control treatments could be calculated. Best, worst, and average effects were calculated for the two treatments, with various possible between-treatment-group effects calculated. Estimates for the between-treatment-group effects varied from 0.19 to 1.02 (Cohen's d) [99].	• It should be based on a systematic search of available evidence.• It can be used for any outcome type (including continuous, binary, ordinal, and time-to-event outcomes).• A choice must be made whether an important and/or a realistic difference is sought.• A number of issues need to be considered when assessing an observed difference:○ Is the evidence available directly relevant to the research question at hand (PICOT assessment)?○ Is the existing evidence of a robust nature? Are there multiple studies available, and were they conducted in a methodologically robust manner? What was the risk of bias?○ Is the outcome of interest fully reported? Individual patient data are seldom available, and reporting of outcomes is often selective.• Determination of a realistic (target) difference can, and when possible should, be based on a systematic review and associated meta-analysis of RCTs, although imprecision in the estimate needs to be considered.• The use of prior evidence can be formalised through simulation of the impact of a new study on the meta-analysis result, although this implies that a particular analysis will be conducted and the new study will be analysed alongside the current evidence.
**Standardised effect size**	Fifty-three nursing home patients received a specialist geriatric medicine consultation. The Goal Attainment Scale was measured post-consultation as part of an observational study. The mean (SD) score was 45.7 (6.9). Using the post-consultation SD and Cohen's criteria, the small, medium, and large effect values were calculated as 1.4, 3.5, and 5.5, respectively [108].	• The SES for a continuous outcome should be calculated as the difference between groups divided by the appropriate SD. For a parallel group trial, the SD will typically be an estimate of the (common) final group SD, which corresponds to an unadjusted analysis of the final scores; the SD of the within-person change score could be used when an analysis of change scores is planned. The benefit of removing within-person variance, such as through an analysis that adjusts for the baseline value, can also be incorporated when the correlation can be estimated.• A SES from a before-and-after treatment study is unlikely to be representative of that achievable in a treatment study, particularly when two active treatments are compared.• Use of Cohen's criteria of interpretation is difficult to justify, although widespread. Modifications to this effect size scale have been suggested. For example, pragmatic trials are generally accepted to have smaller effects than more efficacy-focused studies. The SES may differ in magnitude between clinical areas and outcomes, and when the standard treatment is very effective.• Changes in the variability (e.g., population spectrum) for a continuous outcome can result in a different standardised effect even though the mean difference remains the same. It is important that an estimate of the variability is also specified and that the sample is similar to the anticipated RCT population. For a binary outcome, the target difference (whether a relative or an absolute difference) should be considered in conjunction with the control group event proportion.• It is most appropriate as a fallback option, if other more context-relevant methods for specifying the target difference cannot be used.

IVF, in vitro fertilisation.

### Anchor Method

Implementation of the anchor method varied greatly [6]–[37]. In its most basic form, the anchor method evaluates the minimal (clinically) important change in score for a particular instrument. This is established by calculating the mean change score (post-intervention minus pre-intervention) for that instrument, among a group of patients for whom it is indicated—via another instrument (the “anchor”)—that a minimum clinically important change has occurred. The anchor instrument, the number of available points on the anchor instrument for response, and the corresponding labelling varied between applications. The anchor instrument was most often a subjective assessment of improvement (e.g., global rating of change), though objective measures of improvement could be used (e.g., a 15-letter change in visual acuity as measured on the Snellen eye chart) [34]. The anchor instrument was usually posed to patients alone [19],[35], though in some cases the clinicians' views alone were used. Older studies tended to use a 15-point Likert scale for the anchor instrument, as suggested by Jaeschke and colleagues [16]; more recent studies tended to use five- or seven-point scales instead. Depending upon the study size and/or clinical context, merging of multiple points on the scale may be required. For example, if a seven-point scale has been used but very few people rate themselves at the extremes of this scale (1 and 7), it may be possible to merge points 1 and 2 of the scale and points 6 and 7. It should be noted that it may not always be appropriate to do this, depending on the clinical question under consideration.

Relative change can be incorporated by comparing those for whom an important change was identified to another patient subset (tested using the same instrument and anchor) who reported no change over time. Another common variation is to consider the percentage change score in the instrument under consideration [33], rather than the absolute score change. Determination of what constituted an important difference was sometimes based upon the use of methodology more typically used to assess diagnostic accuracy, such as receiver operating characteristic curves [6],[11],[20], or more complex statistical approaches. It is worth noting that the anchor method was not always successful in deriving values for an important difference; failure was usually due to either practical or methodological difficulties [17],[23].

A substantially different way of achieving an anchor-based approach for specifying an important difference was proposed by Redelmeier and colleagues [28]: in this study, other patients formed a reference against which a patient could rate their own health (or health improvement) [10],[27]–[30]. Generalisability of the resulting estimate of an important difference is a key concern. For example, if the disease is chronic and progressive, an important change value from a newly diagnosed population may not apply to a population with a far longer duration of illness [15],[24],[25],[32],[36]. A key consideration is how to decide on an appropriate cutoff point for the anchor “transition” tool.

Participant biases, such as recall bias, are also potentially problematic [13],[14],[21],[22],[25], as are response shift (whereby patients' perceptions of acceptable change alter during the course of disease or treatment and become inconsistent) [37] and gratitude factor or halo bias (whereby responses that are more favourable than is realistic need to be taken into account) [31],[35]. Another key choice is whether to consider improvement and deterioration together or separately. If a Likert scale has been used as the anchor, improvement and deterioration can be merged to obtain one more general measure for “change” by “folding” the scale at zero, though this assumes symmetry of effect, with “no change” centred upon zero difference. This approach may be unrealistic because of response biases and regression to the mean, and is inappropriate if patients are likely to rate improvements in their health differently from how they would rate deterioration with the same condition. The method proposed by Redelmeier and colleagues, where other participants act as the anchor, avoids recall bias because all data can be collected at the same time, though it may not be a universally appropriate method, as participants might find it difficult to discuss particularly sensitive or private health issues with others.

### Distribution Method

Three distinct distribution approaches were found [38]–[56]: measurement error, statistical test, and rule of thumb. The measurement error approach determines a value that is larger than the inherent imprecision in the measurement and that is therefore likely to be consistently noticed by patients. The most common approach for determining this value was based upon the standard error of measurement (SEM). The SEM can be defined in various ways, with different multiplicative factors suggested as signifying a non-trivial (important) difference.

The most commonly used alternative to the SEM method (although it can be thought of as an extension of this approach) was the reliable change index proposed by Jacobson and Truax [47], which incorporates confidence around the measurement error. For the statistical test approach, a “minimal detectable difference”—the smallest difference that could be statistically detected for a given sample size—is calculated. This is then used as a guide for interpreting the presence of an “important” difference in this study. The rule-of-thumb approach defines an important difference based on the distribution of the outcome, such as using a substantial fraction of the possible range without further justification (e.g., 10 mm on a 100-mm visual analogue scale measuring symptom severity being viewed as a substantial shift in outcome response) [54].

Measurement error and rule-of-thumb approaches are widely used, but do not translate straightforwardly to an RCT target difference. This is because for measurement error approaches, assessment is typically based on test–retest (within-person) data, whereas many trials are of parallel group (between-person) design. Additionally, measurement error is not suitable as the sole basis for determining the importance of a particular target difference. More generally, the setting and timing of data collection may also be important to the calculation of measurement error (e.g., results may vary between pre- and post-treatment) [52]. The statistical test approach cannot be used to specify a priori a target difference in an RCT sample size calculation, as the observed precision of the statistical test is conditional on the sample size. Rule-of-thumb approaches are dependent upon the outcome having inherent value (e.g., Glasgow coma scale), where a substantial fraction of a unit change (e.g., one-third or one-half) can be viewed as important.

### Health Economic Method

The approaches included under the health economic method typically involve defining a threshold value for the cost of a unit of health effect that a decision-maker is willing to pay and using this threshold to construct a “net benefit” that combines both resource cost and health outcomes [57]–[65]. The extent to which data on the differences in costs, benefits, and harms are used depends on the decision and perspective adopted (e.g., treatment *x* is better than treatment *y* when the net benefit for *x* is greater than that for *y,* i.e., the incremental net benefit for *x* compared to *y* is positive) [62]. The net benefit approach can be extended into a decision-theoretic model in order to undertake a value of information analysis [60],[61],[65], which seeks to address the value of removing the current uncertainty regarding the choice of treatment. The optimal sample size of a new study given the current evidence and the decision faced can be calculated. The perspective of the decision-making is critical, i.e., whether it is from the standpoint of clinicians, patients, funders, policy-makers, or some combination.

More sophisticated modelling approaches can potentially allow a comprehensive evaluation of the treatment decision and the potential value of a new study, though they require strong assumptions about, for example, different measurements of effectiveness, harms, uptake, adherence, costs of interventions, and the cost of new research. The increased complexity, along with the gap between the input requirements of the more sophisticated modelling approaches and the data that are typically available, and the need to be explicit about the basis of synthesis of all the evidence upfront, perhaps explains the limited use of these modelling approaches in practice to date.

### Opinion-Seeking Method

The opinion-seeking method determines a value (or a plausible range of values) for the target difference, by asking one or more individuals to state their view on what value or values for a particular difference should be important and/or realistic [66]–[86]. The identified studies varied widely in whose opinion was sought (e.g., patients, clinicians, or trialists), the method of selecting individual experts (e.g., literature search, mailing list, or conference attendance), and the number of experts consulted. Other variations included the method used to elicit values (e.g., interview or survey), the complexity of the data elicited, and the method used to consolidate results into an overall value or range of values for the difference.

One advantage of the opinion-seeking method is the ease with which it can be carried out (e.g., through a survey). However, estimates will vary according to the specified population. Additionally, different perspectives (e.g., patient versus health professional) may lead to very different estimates of what is important and/or realistic [73]. Also, the views of approached individuals may not necessarily be representative of the wider community. Furthermore, some methods for eliciting opinions have feasibility constraints (e.g., face-to-face methods), but alternative approaches for capturing the views of a larger number of experts require careful planning or may be subject to low response rates or partial responses [77].

### Pilot Study Method

A small number of studies used a pilot study method to determine a relevant value for the target difference [87]–[90]. A pilot study can be defined as running the intended study in miniature prior to conducting the actual trial, to guide expectations on an appropriate value for the target difference. The simplest approach is to use the observed effect in the pilot study as the target difference in an RCT. More sophisticated approaches account for imprecision in the estimate from the pilot study and/or use the pilot study to estimate only the standard deviation (SD) (or control group event proportion) and not the target difference.

However, there are practical difficulties in conducting a pilot study that may limit the relevance of results [87], most notably the inherent uncertainty in results due to the small study sample size, rendering the effect size imprecise and unreliable. Additionally, a pilot study can address only a realistic difference and does not inform what an important difference would be. Finally, it is worth noting that an internal pilot study, using the initial recruits within a larger study, cannot be used to pre-specify the target difference, though it could inform an adaptive update [90]. Notwithstanding the above critique, a pilot study can have a valuable role in addressing feasibility issues (e.g., recruitment challenges) that may need to be considered in a larger trial [89]. Pilot studies are most useful when they can be readily and quickly conducted. While few studies addressed using a pilot study to inform the specification of the target difference, trialists may use pilot studies to help determine the target difference without reporting this formally in trial reports.

### Review of the Evidence Base Method

Implementation of the review of the evidence base method varied regarding what studies and results were considered as part of the review and how the findings of different studies were combined [91]–[103]. The most common approach involved implementing a pre-specified strategy for reviewing the evidence base for either a particular instrument or variety of instruments to identify an important difference. Alternatively, pre-existing studies for a specific research question may be used (e.g., using the pooled estimate of a meta-analysis) to determine the target difference [100]. Extending this general approach, Sutton and colleagues [101] derived a distribution for the effect of treatment from the meta-analysis, from which they then simulated the effect of a “new” study; the result of this study was added to the existing meta-analysis data, which were then re-analysed. Implicitly this adopts a realistic difference as the basis for the target difference.

Reviewing the existing evidence base is valuable as it provides a rationale for choosing an important and/or realistic target difference. It is likely that this general approach is often informally used, though few have addressed how it should be formally done. However, estimates identified from existing evidence may not necessarily be appropriate for the population being considered for the trial, so the generalisability of the available studies and susceptibility to bias should be considered. For reviews of studies that identified an important difference, the methods used in each of the individual studies to determine that difference are subject to the practical issues mentioned here for that method (e.g., the anchor method). Imprecision of the estimate is also an important consideration, and publication bias may also be an issue if reviews of the evidence base consider only published data. If a meta-analysis of previous results is used to determine a sample size, then additional evidence published after the search used in the meta-analysis was conducted may necessitate updating the sample size.

### Standardised Effect Size Method

This method is commonly used to determine the importance of a difference in an outcome when set in comparison to other possible effect sizes upon a standardised scale [88],[104]–[116]. Overwhelmingly, studies used the guidelines suggested by Cohen [106] for the Cohen's d metric, i.e., 0.2, 0.5, and 0.8 for small, medium, and large effects, respectively, in the context of a continuous outcome. Other SES metrics exist for continuous (e.g., Dunlap's d), binary (e.g., odds ratio), and survival (hazard ratio) outcomes [106],[111],[116]. Most of the literature relates to within-group SESs for a continuous outcome. The SD used should reflect the anticipated RCT population as far as possible.

The main benefit of using a SES method is that it can be readily calculated and compared across different outcomes, conditions, studies, settings, and people; all differences are translated into a common metric. It is also easy to calculate the SES from existing evidence if studies have reported sufficient information. The Cohen guidelines for small, medium, and large effects can be converted into equivalent values for other binary metrics (e.g., 1.44, 2.48, and 4.27, respectively, for odds ratio) [105]. As noted above, SES metrics are commonly used for binary (e.g., odds ratio or risk ratio) and survival outcomes (e.g., hazard ratio) in medical research [111], and a similar approach can be readily adopted for such outcomes. However, no equivalent guideline values are in widespread use. Informally, a doubling or halving of a ratio is sometimes seen as a marker of a large relative effect [109].

It is important to note that SES values are not uniquely defined, and different combinations of values on the original scale can produce the same SES value. For the standard Cohen's d statistic, different combinations of mean and SD values produce the same SES estimate. For example, a mean (SD) of 5 (10) and 2 (4) both give a standardised effect of 0.5SD. As a consequence, specifying the target difference as a SES alone, though sufficient in terms of sample size calculation, can be viewed as insufficient in that it does not actually define the target difference for the outcome measure of interest. A limitation of the SES is the difficulty in determining why different effect sizes are seen in different studies: for example, whether these differences are due to differences in the outcome measure, intervention, settings, or participants in the studies, or study methodology.

### Combining Methods

The vast majority of studies that combined methods used two or three of the anchor, distribution, and SES methods. Studies that used multiple methods were not always clear in describing whether and how results were triangulated, and for certain combinations the result of one method seemed to be considered of greater value than the result of another method (i.e., as if a primary and supplementary method had been selected). For example, values that were found using the anchor method were often chosen over effect size results or distribution-based estimates [117]. Alternatively, the most conservative value was chosen, regardless of the comparative robustness of the methods used [118]. In cases where the results of the different methods were similar, triangulation of the results was straightforward [119].

## Discussion

This comprehensive systematic review summarizes approaches for specifying the target difference in a RCT sample size calculation. Of the seven identified methods, the anchor, distribution, and SES methods were most widely used. There are several reasons for the popularity of these methods, including ease of use, usefulness in studies validating quality of life instruments, and simplicity of calculation of distribution and SES estimates alongside the anchor method. While most studies adopted (though typically implicitly) the conventional Neyman-Pearson statistical framework, some of the methods (i.e., health economic and opinion-seeking) particularly suit a Bayesian framework.

No further methods were identified by this review beyond the seven methods pre-identified from a scoping search. However, substantial variations in implementation were noted, even for relatively simple approaches such as the anchor method, and many studies used multiple methods. Most studies focused on continuous outcomes, although other outcome types were considered using opinion-seeking and evidence base review. While the methods could in principle be used for any type of RCT, they are most relevant to the design of Phase 3, or “definitive”, trials.

A number of key issues were common across the methods. First, it is critical to decide whether the focus is to determine an important and/or a realistic difference. Some methods can be used for both (e.g., opinion-seeking), and some for only one or the other (e.g., the anchor method to determine an important difference and the pilot study method to determine a realistic difference). Evaluating how the difference was determined and the context of determining the target difference is important. Some approaches commonly used for specifying an important difference either cannot be used for specifying a target difference (such as the statistical test approach) or do not straightforwardly translate into the typical RCT context (for example the measurement error approach). The anchor, opinion-seeking, and health economic methods explicitly involve judgment, and the perspective taken in the study is a key consideration regarding their use. As a consequence, these methods explicitly allow different perspectives to be considered, and in particular enable the views of patients and the public to be part of the decision-making process.

Some methodological issues are specific to particular methods. For example, the necessity of choosing a cutoff point to define an “important” difference/change is specific to the anchor method. This approach is a widely recognised part of the validation process for new quality of life instruments, where the scale has no inherent meaning without reference to an outside marker (i.e., anchor).

All three approaches of the distribution method—measurement error, statistical test, and rule of thumb—have clear limitations, the foremost being that they do not match the setting of a standard RCT design (two parallel groups). The statistical test approach cannot be used to specify a target difference, given that it is essentially a rearranged sample size formula. The rule-of-thumb approach is dependent upon the interpretability of the individual scale.

The SES method was used in a substantial number of studies for a continuous outcome, but was rarely reported for non-continuous outcomes, despite informal use of such an approach probably being widespread. No parallel for a binary outcome exists, though odds ratio values approximately equivalent to Cohen's d values can be used. The validity of Cohen's cutoffs is uncertain (despite widespread usage), and some modifications to the original values have been proposed [120],[121].

The opinion-seeking method was often used with multiple strategies involved in the process (e.g., questionnaires being sent to experts using particular sampling methods, followed by an additional conference being organised to discuss findings in more detail). The Delphi technique for survey development and the nominal group technique for face-to-face meetings are commonly used and are potentially useful for this type of research when developing instruments. In terms of planning a trial, the opinion-seeking method can be relatively easy to implement, but the resulting usefulness of the estimated target difference may depend on the robustness of the approach used to elicit opinions.

The health economic and pilot study methods were infrequently reported as specific methods. For the health economic method, this is likely due to the complexity of the method and/or the resource-intensive procedures that are required to conduct the theoretically more robust variants that have been developed. The use of pilot studies to determine the target difference is problematic and probably only useful for the control group event proportion or SD, for a binary or continuous outcome, respectively. Internal pilot studies may be incorporated into the start of larger clinical trials, but are not useful for specifying the target difference, though they could be used to revise the sample size calculation. The review of the evidence base method can be applied to identify both an important or realistic difference; a pilot study addresses only a realistic difference. For both methods, applicability to the anticipated study and the impact of statistical uncertainty on estimates should be considered.

A review of the evidence base approach for a particular outcome measurement or study population may be combined with any of the other methods identified for establishing an important difference. However, the number of studies reporting a formal method for identifying an important difference using the existing evidence was surprisingly small. It could be that there is wide variation in the extent to which reviews of the existing evidence base have been undertaken prospectively using a specific and formal strategy.

Some methods can be readily used with others, potentially increasing the robustness of their findings. The anchor and distribution methods were often used together within the same study, frequently also with the SES approach. Multiple methods for specifying an important difference were used in some studies, though the combinations varied, as did the extent to which results were triangulated. The result of one method may validate the result found using another method, but conflicting estimates increase uncertainty over the estimate of an important difference.

### Strengths and Limitations

To our knowledge, this review is the first comprehensive and systematic search of all possible methods for specifying a target difference. The search strategy was inclusive, robust, and logical; however, this led to a large number of studies that did not report a method for specifying an important and/or realistic difference. Also, it is possible some studies were missed because of the lack of standardised terminology. Finally, our search period ended in January 2011, and another method not included in the seven identified by this review may have been published since then, although we believe this is unlikely. More likely is the use of new variations in the implementation of existing methods.

### Conclusions

A variety of methods are available that researchers can use for specifying the target difference in an RCT sample size calculation. Appropriate methods and implementation vary according to the aim (e.g., specifying an important difference versus a realistic difference), context (research question and availability of data), and underlying framework adopted (Bayesian versus conventional statistical approach). No single method provides a perfect solution for all contexts. Some methods for specifying an important difference (e.g., a statistical test–based approach) are inappropriate in the RCT sample size context. Further research is required to determine the best uses of some methods, particularly the health economic, opinion-seeking, pilot study, and SES methods. Prospective comparisons of methods in the context of RCT design may also be useful. Better reporting of the basis upon which the target difference was determined is needed [122].

## Supporting Information

Checklist S1 **PRISMA checklist.**
(DOC)

Protocol S1 **Systematic review protocol.**
(DOC)

Search Strategy S1 **Systematic review search strategy.**
(DOCX)

## Abbreviations

ICH: International Conference on Harmonisation of Technical Requirements for Registration of Pharmaceuticals for Human Use
RCT: randomised controlled trial
SD: standard deviation
SEM: standard error of measurement
SES: standardised effect size
