# Supplementary material for: Methods for Specifying the Target Difference in a Randomised Controlled Trial: The Difference ELicitation in TriAls (DELTA) Systematic Review
Source: PLoS Med. 2014 May 13;11(5):e1001645. doi: 10.1371/journal.pmed.1001645 (PMC4019477; doi:10.1371/journal.pmed.1001645)
Supplement: Protocol S1 — Systematic review protocol. (DOC) [file pmed.1001645.s002.doc]

**
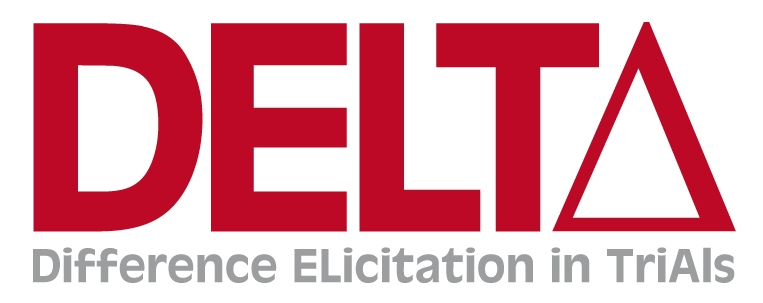
**

**Assessing methods to specify the target difference for a randomised controlled trial (Difference ELicitation in TriAls – DELTA review)**

**This study was part of a project commissioned and funded by the UK Medical Research Council & National Institute for Health Research Joint Methodology Research Programme (MRC005885 & G0902147).**

**Background on targeted differences:**

**Calculation of sample size**

The randomised controlled trial (RCT) is widely considered to be the gold standard for the comparison of the effectiveness of health interventions.^1^ Central to its validity is an *a priori* sample size calculation which sets the recruitment target for a particular study, which (assuming the target recruitment is reached) provides reassurance that the trial result will be informative because it is likely to detect a difference with the appropriate level of statistical precision.

To calculate the sample size for a superiority trial, a compromise is required between the possibility of being mislead by chance and the risk of not identifying a genuine difference. Rejecting the null hypothesis when it is true (Type I error) would lead to a trial concluding that one treatment is superior than another when in reality there is no significant difference in treatment effect. The significance level of the test (α) is the probability of the occurrence of Type I error. Failing to reject the null hypothesis when it is false (Type II error) would lead to a trial concluding that one treatment is not superior to another, when in reality that treatment is superior. The probability of the occurrence of Type II error is 1 minus the power of the test, or put another way, the power of the test is denoted by 1 – β, where β is the probability of Type II error. Commonly used values are 0.01 or 0.05 for a type I error (the statistical significance level) and 0.1 or 0.2 for a type II error (which would. give 90% or 80% power to detect a difference of the size specified) under the conventional (Neyman-Pearson) statistical approach.^2^ Once these two criteria are set, and the statistical tests to be conducted during the analysis stage are chosen, the sample size is determined depending on the magnitude of difference to be detected. This “targeted” difference, is the magnitude of difference which the RCT is designed to reliably investigate.

For equivalence (or non-inferiority) trials, as opposed to superiority trials, a range of values around zero will be required within which the interventions are deemed to be effectively equivalent (or not inferior), in order to establish the magnitude of difference that the RCT is designed to investigate. The limits of this range are points at which the differences between treatments are believed to become important and one of the treatments is considered superior: the smallest difference between one of these points and zero is the minimum important difference between treatments.

Once the target difference (or in the case of an equivalence/inferiority trial limits) is determined then the method of estimating the sample size will depend upon the proposed statistical analysis, trial design (e.g. cluster randomised or individual randomisation trial) and statistical properties specified (e.g. agreement for paired data). The general approach is similar across studies under the standard Neyman-Pearson. Other statistical approaches for defining the required sample size are Fisherian, Bayesian and decision-theoretic Bayesian approaches, along with a hybrid of both the Bayesian and Neyman-Pearson approaches.^3^ Economic-based methods tend to follow a Bayesian approach. However, a recent review of RCT sample size calculations identified only the Neyman-Pearson approach in widespread usage.^4^ Regardless of the statistical method used the key issue is what magnitude of a difference (given it is statistically detected) is of practical interest.

**The target difference**

From both a scientific and ethical standpoint, selecting an appropriate target difference is of crucial importance. For example, two drugs for treating hypertension may differ in how well they reduce blood pressure. A small difference based upon blood pressure may have limited clinical, patient or economic significance. As a consequence, specifying too small a target difference would be a wasteful (and unethical) use of data and resources. Conversely, too large a target difference could lead to an important difference being easily overlooked, which could also be a wasteful (and unethical) use of data and resources. Furthermore, an undersized study may not usefully contribute to the knowledge base and could detrimentally impact upon decision making.^5^

An important development has been the concept of the minimum clinically important difference (MCID) as a rationale to define the target difference. Originally, this was defined as the “the smallest difference …. which patients perceive as beneficial and which would mandate, in the absence of troublesome side effects and excessive cost, a change in the patient’s management.”^6^, but has also been referred to as the “minimum difference that is important to a patient”.^7^ The concept applies equally well to the minimum difference that is important to a clinician, or to society, though obviously there could be variations between these groups (patient, clinician and society) in terms of what magnitude of a difference each considers to be important. A clinician might consider it to be the difference that would result in a change of treatment strategy for the patient, society might consider it to be the difference that would result in a return to productive employment for the patient whereas a patient might consider it any difference that they are personally aware of.^8^

Variations have been suggested such as the “minimally clinically important improvement” and the “sufficiently important difference”, which seeks to adopt a wider perspective by taking into account cost, risk and harms.^9, 10, 11^ In fact, a variety of economic approaches have been suggested from both a conventional and Bayesian perspective.^11 12^ All seek to ascertain a cut-point for a scale (whether directly measureable or latent) upon which an “important” difference or change can be separated from an “unimportant” one. Most work has been carried out on patient reported outcomes, (reflecting the belief that patients find it more difficult than clinicians to specify an important difference) and also the challenge of interpreting quality of life measures.^6, 13^ In addition, there are pragmatic challenges in the interpretation of a mean value for the minimum important difference, where this value is the average change in score derived from all patients who have experienced what they would classify as an important change. Using a mean value will classify those with a change score below the mean as not having experienced an important change. In reality however all patients had experienced what they would classify as an important change. Selecting cut-offs for individuals has been suggested as a possible solution, but more generally it has been argued that the interpretation of important change needs to be considered differently when considering individuals or considering groups.^8^

In practice, the target difference is often not formally based upon these concepts and in many cases (at least from trial reports) appears to be determined upon convenience or some other informal basis.^14^ A variety of methods have been proposed to formally determine a target difference (including those for the MCID and its variants).^7, 11^

**Existing methods for specifying target differences**

There are at least six main formal approaches to identify a targeted difference upon which to base the sample size calculation:

1. *Opinion seeking methods:*

Formal methods for determining the target difference on the basis of eliciting expert opinion (usually clinicians) have been proposed through organisation of either a conference of experts, surveys of members of professional bodies or regulatory committees, or individual interviews.^15^ A formal Bayesian elicitation enabling both expectation of the difference and also a range of values (for which the trial result could be categorised as one or the other of the treatments being superior or equivalent) has been used.^16^ Clinical judgement may identify interventions which are expected to be similar and for which empirical evidence is available (see method 5 below). Informally, clinical opinion will always be one of the aspects considered.

1. *Distribution methods:*

Such methods typically determine a value that is larger than the inherent imprecision in the measurement and therefore likely to represent a meaningful difference (this includes determining smallest/minimally detectable change). Other methods are based upon the nature of the outcome (e.g. a fraction of the response range for a visual analogue scale).

1. *Standardised effect size approach:*

Under such an approach, the statistical characteristic of the outcome measure is used to define the target difference. For a continuous outcome, the standardised difference (most commonly expressed as Cohen’s “effect size”) can be used. Interpretation of the "effect size” approach is heavily reliant on the work by Cohen^17^ in giving values of 0.2, 0.5 and 0.8 for small, medium and large effects. Alternatively, the measurement error associated with the outcome can be accounted for (e.g. based upon test-retest reliability) to provide a value that can be characterised as a non-spurious difference. For a binary or time-to-event outcome, risk or hazard ratios respectively, could be utilised. As for the standardised mean difference, an interpretation can be applied to the spectrum of values.

1. *Anchor-based methods:*

Under such methods a difference in a outcome measure can be defined as signifying a “change” in status by asking an assessor to judge whether a change (beneficial or otherwise) has occurred. Commonly, patients assess their own change (e.g. before versus after treatment). The values associated with those experiencing a change would then be used to determine the magnitude of difference in the outcome which signifies an important difference. From this the target difference is determined. Variations exist in terms of who assesses change (e.g. patient or clinician), how they assess change (e.g. a change and/or an important changes), what they compare against (e.g. before and after treatment or another patient with the same condition) and how the responses are summarised (e.g. the mean value or receiver operating curve cut-point determination). Rarely have the methods been used and set up with a RCT specifically in mind.^11^ A Bayesian application could use prior information to determine the size needed for the posterior distribution to rule in or rule out an important difference.

1. *Commissioned research:*

A preliminary or pilot study may be carried out where there is little evidence, or even experience, to guide expectations. A pilot study provides support for estimates where relatively small misspecifications could have substantial impact upon corresponding precision and the power to detect a difference (e.g. screening trial).^18^

1. *Review of evidence base:*

The target difference can be derived using current evidence. Ideally this would be based upon a systematic review of RCTs, and possibly meta-analysis, of the outcome(s) of interest directly addressing the research question at hand. In the absence of randomised evidence, observational evidence has been used in a similar manner. Trials are based upon what the current evidence base suggests is plausible for the parameters of interest. Conventionally, studies are powered in isolation from any current evidence but a formal meta-analysis sample size approach could allow previous evidence to be incorporated in a power calculation akin to the Bayesian Neyman-Pearson hybrid approach.^19^

1. *Health Economic approaches:*

Recent approaches have used the net monetary benefit (NMB) statistic to define a target difference.^12,20,21^ An intervention is considered efficient if the NMB for an intervention compared with a comparator is greater than zero (or that the likelihood that the NMB is greater than zero is acceptable). The Bayesian expected value of sampling information approach weighs the expected benefits provided by new research against the expected costs of this research for the key parameters, determining the cost-effectiveness. A distribution for the target difference in a parameter or group of parameters can be inferred.

**Aims and Objectives**:

The aim of this project is to consider all potentially relevant methods of defining a target difference in order to develop clear guidance for researchers on appropriate methods to use under varying circumstances.

To achieve this aim, the following are key objectives for the project:

1. To conduct a systematic review of the methods for identifying a target difference (that has been developed either within or outside the health field), critically appraising the usefulness of each method for different types of RCTs.
2. To identify the methods currently considered as “best” practice using a survey of UK and Ireland based Clinical Trial Units, MRC Trial Hubs and the membership of the Society of Clinical Trials.
3. To develop draft guidance to be discussed in a workshop and symposium, and incorporate feedback from these events into finalised guidance for researchers.
4. To identify future research needs

An expert Advisory Group will monitor and review the progress of the project along with the Project Steering Group (Grantholders and lead project research fellow). In this abridged version of the protocol, the methodology of the systematic review of methods for identifying a target difference is reported. Methodology relating to the research to address objectives 2-4 is not included.

**Methods:**

*Search Strategy*

The search strategy will involve conducting an electronic literature search of both biomedical and some non-biomedical databases (e.g. Econlit), building on preliminary work already undertaken as part of the original grant application process (see Appendix 1 for example search).

It is proposed that both biomedical and some non-biomedical databases are searched, as studies using methods that may be of relevance to RCTs can often be performed in other fields (e.g. behavioural sciences), and restriction to the biomedical field may miss ‘novel’ methods of relevance to the biomedical field. Relevant literature from biomedicine, the social sciences and science and technology fields will therefore be searched, and the databases to be considered include:

- MEDLINE
- EMBASE
- CENTRAL
- Cochrane Methodology Register
- Science Citation index (SCI)
- Econlit
- PsycINFO
- Education Resources Information Centre (ERIC)

There will be no language restriction and searches will be undertaken on dates from 1966 onwards (or from the start of database coverage). A limit on the number of databases searched may be required if the number of records identified is particularly high.

The search of electronic databases will also be augmented in other ways including:

1. Cited reference search
   - The Web of Science and Scopus databases will be used to identify studies referencing any of the key methodological papers that have already been identified through the main electronic database search.
2. Reference lists of included studies
   - These will be checked to identify new methods or variants of a known method.
3. Hand searching
   - The relevant literature is likely to be distributed across many fields and journals. If a particular journal is the source of many papers, hand-searching of this journal may be considered. However, this method is resource-intensive and the extent to which it will be undertaken depends on the resources available following completion of higher priority tasks. In addition to hand searching journals, standard clinical trial text books will be reviewed to ascertain if they reference or describe a method for eliciting a target difference. General clinical trials books or books on calculating the sample size for clinical trial published in the last 5 years will be reviewed. Additionally, older textbook that are viewed as influential in the trial community will be also reviewed.
4. Contacting those with an interest in the field
   - A number of key figures are involved in this project as either applicants or named collaborators. In addition, authors of key studies identified may be contacted for information on additional available evidence.
5. Grey literature searching
   - Guidance documents from regulatory authorities (e.g. FDA) and known international standards organisations (e.g. International Conference on Harmonisation of technical requirements for registration of pharmaceuticals for human use) will be searched.
6. Methods being used by UK Clinical researchers
   - Information on methods currently in use will be identified from the surveys (see Objective 2 for more details).

An exploration of best combinations of subject headings and text word searching (searching in titles, abstracts and author specified keywords) will be undertaken before full searches are carried out. It is anticipated that few indexed terms will be suitable and therefore the search strategies are expected to mainly consist of text words and phrases using appropriate synonyms, truncation symbols and adjacency operators.

*Inclusion and Exclusion Criteria*

This review will concentrate on papers identifying new methods (or a significant variant of established methods) for determining the target difference, although established methods will be referenced and are likely to be identified by the search strategy. Papers published, in any language, will be included which specify methods for determining the target difference (either explicitly or implicitly).

It is likely that a variety of methods will meet our inclusion criteria, and although they should be relevant to RCTs, they may not be reported in RCTs themselves or necessarily used in this context. As the focus of the review is to identify methods for establishing target differences, we will not restrict inclusion depending on the type of study design (e.g. RCT, quasi-experimental, etc.) in which a method may have been applied will be made. Where a method has been identified from a report of a primary study, details of the type of primary study from which it came will be noted at data extraction stage. In terms of outcomes all types of outcome (e.g. dichotomous, continuous) relevant to clinical trials, including efficacy, effectiveness and cost-effectiveness ones will be eligible. Included papers will have to report a real or hypothetical example which seeks to determine a difference (explicitly or implicitly via providing a basis for study size), based on at least one outcome of relevance to clinical trials or which could be used for this purpose.

Inclusion criteria are:

- Reporting a method which could be used to specify a target difference. A method may implicitly specify the target difference by determining the optimal study sample size. The assessment must be based on at least one outcome of relevance to clinical trials or could be used for this purpose. The use of a method in a hypothetical scenario is eligible for inclusion.

Exclusion criteria are:

- Studies failing to report a method for specifying a target difference.
- Systematic reviews of methods for specifying the target difference or of outcome scales. These reports will be retained and their reference lists reviewed for potentially eligible studies. Such papers will only be included if it is the primary reference for a relevant method.
- Studies reporting only on the statistical considerations for sample size (e.g. a new formula for the sample size calculation) will not be considered sufficient to meet the inclusion criteria.
- Studies which discuss a metric (e.g. risk ratio or number needed to treat) without reference to how a specific difference could be determined will not be considered relevant for inclusion.

It is anticipated that it will not be possible during the initial abstract screening phase, to exclude or include with absolute certainty, potentially relevant papers reporting target difference methods. This is expected because abstracts are brief summaries and relevant information on the method used to elicit a target difference may not be sufficient within an abstract to allow a final decision on inclusion to be made. In addition, it is likely that many papers reporting methods for eliciting target difference may report the use of existing methods rather than new methods (or substantial variation of existing methods) not previously identified. As a result, many papers may report the same method. It will therefore be useful to provisionally categorise papers by their abstracts at the review screening stages, in order to split included papers depending on the method reported, prior to extracting more detailed information (e.g. outcome measures) at the full-text data extraction stage.

Titles and abstracts of the search strategy results will be screened by one reviewer in the first instance, but where there is uncertainty the opinion of a second reviewer will be used, and if necessary a third member of the team will act as an arbiter where there is disagreement. Full text papers will be obtained where, on initial screening of the abstract, the work is considered to be potentially relevant and these papers will be assessed to confirm inclusion or exclusion in the review.

A register of studies meeting the inclusion criteria will be organised using Reference Manager bibliographic software using the key word facility to classify articles by type, reference source and methodology.

*Data Extraction Strategy*

At the screening stages of the review, included (or potentially relevant) papers will be categorised by the method they report. Following the categorisation of papers by the method reported, data will be extracted from papers to help summarise the variation and range of applicability of each method. Data extracted at this stage will include (where reported):

1. What is measured

e.g. single measure of clinical effectiveness or safety, composite measure of clinical effectiveness and/or safety, a measure of overall (or disease specific) health, or cost/cost-effectiveness measure.

2) Type of outcome measure

e.g. binary, ordinal, continuous or time-to-event

3) Relevant summary measure reported

e.g. risk ratio, absolute risk difference, mean difference

4) Size of the sample used to elicit value for important difference (where reported)

5) Perspective used to define target difference

e.g. patients’ and clinicians’

Data will also be extracted on the following details (where they have been reported):

- The context in which the difference was elicited (e.g. real or hypothetical RCT)
- Terminology used to describe the important difference
- Methodological details and noteworthy features (e.g. unique variations)

It may be necessary to extract different information depending on the method used. For example, details of any formulas used for distribution methods are unlikely to apply to expert opinion methods. As a result, the common factors listed above will be extracted along with specific information relevant to each particular method, and therefore no generic data extraction form will be used.

*Method of Analysis*

A summary description of each method found will be produced by reporting extracted details (and also any slight variants of the method).The key characteristics of each method will be categorised. A cube classification system for studies of responsiveness has been proposed.^11^ However, some modification/or simplifications to the original method may be necessary when applied to the available evidence, particularly in considering the implications of applying markedly different methods to RCTs.

Once the methods have been classified and the evidence available summarised, an assessment of the strengths and weaknesses of each method will be undertaken. This will link the review of the available evidence to the development of methodological guidance for eliciting targeted differences (see Objective 3). The criteria used to critique all methods will be developed in relation to the key focus of this review (i.e. applicability of methods to a clinical trial setting) and may include aspects such as the practical feasibility of using the method, the appropriateness of the method, its reliability and validity etc).The critique will also evaluate whether particular methods are better suited to particular stages of development of an intervention. Uncertainty will be considered with regard to both the calculation of precision around the proposed target difference found using each method, and the extent to which the target difference value can account for, or might be modified by, other potential outcome measures for the same study population being considered.

The criteria itself will be developed and finalised in consultation and agreement with the project steering and advisory groups. Existing checklists that have been developed for other methodological reviews may also be used to help develop the criteria to be used to assess the strengths and weaknesses of each method being considered by this review, although it is likely that no existing checklist will be fully relevant or applicable to this review.

**Project Members**

**Steering Group Members:**

Dr Jonathan Cook

Professor Luke Vale

Ms Jenni Hislop

Ms Cynthia Fraser

Dr Craig Ramsay

Professor Peter Fayers

Professor Andrew Briggs

Professor John Norrie

Professor Doug Altman

Professor Ian Harvey

Dr Brian Buckley

**Advisory Committee Members:**

Professor Marion Campbell

Professor Adrian Grant

Professor Ian Ford

Professor George Wells

Professor Dean Fergusson

**Competing Interests of Members:**

None stated

**References**

1. Altman DG, Schulz KF, Moher D, Egger M, Davidoff F, Elbourne D et al. The revised CONSORT statement for reporting randomized trials: explanation and elaboration. *Ann Intern Med* 2001;134:663-94.
2. Pocock SJ. Clinical Trials – A practical approach. 1996 John Wiley & Sons Ltd.
3. Spiegelhalter DJ, Abrams KR, Myles JP. Bayesian approaches to clinical trials and health-care evaluation. Chichester: John Wiley & Sons; 2003.
4. Charles P, Giraudeau B, Dechartres A, Baron G, Ravaud P. Reporting of sample size calculation in randomised controlled trials: review. *BMJ 338:b1732,* 2009
5. Fayers PM and Machin D. Sample size: how many patients are necessary? *British Journal of Cancer* 1995;72:1-9
6. Jaeschke R, Singer J, Guyatt GH. Measurement of health status. Ascertaining the minimal clinically important difference. *Control Clin Trials* 1989;10:407-15
7. Copay AG, Subach BR, Glassman SD, Polly J, Schuler TC. Understanding the minimum clinically important difference: a review of concepts and methods. *Spine Journal* 2007;7:541-6
8. Beaton DE, Boers M, Wells GA. Many faces of the minimal clinically important difference (MICD): A literature review and directions for future research. *Curr Opin Rheumatol* 2002;14:109-14
9. Kvien TK, Heiberg T, Hagen KB. Minimum clinically important improvement/difference (MCII/MCID) and patient acceptable symptom state (PASS): what do these concepts mean? *Ann Rheum Dis* 2007;66(Suppl III):iii40-iii41
10. Barrett B, Brown D, Mundt M, Brown R. Sufficiently important difference: expanding the framework of clinical significance. *Med Decis Making* 2005;25:250-61.
11. Wells G, Beaton D, Shea B, Boers M, Simon L, Strand V et al. Minimal clinically important differences: Review of methods. *J Rheumatol* 2001;28:406-12.
12. Briggs AH, Gray AM. Power and sample size calculations for stochastic cost-effectiveness analysis. *Med Decis Making* 1998;18:S81-S92
13. Hays RD, Woolley JM. The concept of clinically meaningful difference in health-related quality-of-life research. How meaningful is it? *Pharmacoeconomics* 2000;18:419-23
14. Chan KBY, Man-Son-Hing M, Molnar FJ, Laupacis A. How well is the clinical importance of study results reported? An assessment of randomized controlled trials. *Can Med Assoc J* 2001;165:1197-202.
15. Molnar FJ, Man-Son-Hing M, Fergusson D. Systematic review of measures of clinical significance employed in randomized controlled trials of drugs for dementia. *J Am Geriatr Soc* 2009;57:536-46.
16. Fayers PM, Cuschieri A, Fielding J, Craven J, Uscinska B, Freedman LS. Sample size calculation for clinical trials: the impact of clinician beliefs. *Br J Cancer* 2000;82:213-9.
17. Cohen J. *Statistical power: analysis of behavioural sciences.* New York: Academic Press; 1977.
18. Lancaster GA, Dodd S, Williamson PR. Design and analysis of pilot studies: Recommendations for good practice. *J Eval Clin Pract* 2004;10:307-12
19. Sutton AJ, Cooper NJ, Jones DR, Lambert PC, Thompson JR, Abrams KR. Evidence-based sample size calculations based upon updated meta-analysis. *Stat Med* 2007;26:2479-500
20. O'Hagan A, Stevens JW. Bayesian assessment of sample size for clinical trials of cost-effectiveness. *Med Decis Making* 2001;21:219-30
21. Laska EM, Meisner M, Siegel C. Power and sample size in cost-effectiveness analysis. *Med Decis Making* 1999;19:339-43

**Appendix 1 – Example Strategy [MEDLINE]:**

1 mcid.tw.

2 (target$ adj1 difference?).tw.

3 change score.tw.

4 change point.tw.

5 (clinical$ importan$ adj2 (difference? or change? or improvement? or effect?)).tw

6 (minim$ importan$ adj2 (difference? or change? or improvement? or effect?)).tw.

7 (clinical$ meaningful$ adj2 (difference? or change? or improvement? or effect?)).tw.

8 (minim$ meaningful$ adj2 (difference? or change? or improvement? or effect?)).tw.

9 (smallest meaningful$ adj2 (difference? or change? or improvement? or effect?)).tw.

10 (minim$ significant$ adj2 (difference? or change? or improvement? or effect?)).tw.

11 (smallest significant$ adj2 (difference? or change? or improvement? or effect?)).tw.

12 (minim$ detect$ adj2 (difference? or change? or improvement? or effect?)).tw.

13 (smallest detect$ adj2 (difference? or change? or improvement? or effect?)).tw.

14 (sufficient$ importan$ adj2 (difference? or change? or improvement? or effect?)).tw.

15 (sufficient$ meaningful$ adj2 (difference? or change? or improvement? or effect?)).tw.

16 (minim$ clinical$ adj2 (important or detectable or meaningful)).tw.

17 ((calculat$ or determin$ or comput$) adj2 meaningful).tw.

18 ((calculat$ or determin$ or comput$) adj2 detectable).tw.

19 ((calculat$ or determin$ or comput$) adj2 important adj2 (difference? or change? or improvement? or effect?)).tw.

20 ((calculat$ or determin$ or comput$) adj2 meaningful adj2 (difference? or change? or improvement? or effect?)).tw.

21 ((calculat$ or determin$ or comput$) adj2 detectable adj2 (difference? or change? or improvement? or effect?)).tw.

22 (definition$ adj2 (difference? or change? or improvement?)).tw.

23 *sample size/

24 ((responsiveness adj2 (calculat$ or determine$ or comput$)) and (measure$ or scale$ or score$ or rating$)).tw.

25 or/1-24
