# Supplementary material for: Methods for Specifying the Target Difference in a Randomised Controlled Trial: The Difference ELicitation in TriAls (DELTA) Systematic Review
Source: PLoS Med. 2014 May 13;11(5):e1001645. doi: 10.1371/journal.pmed.1001645 (PMC4019477; doi:10.1371/journal.pmed.1001645)
Supplement: Search Strategy S1 — Systematic review search strategy. (DOCX) [file pmed.1001645.s003.docx]

**Supplementary file 2 – Search Strategies**

**APPENDIX 2 LITERATURE SEARCH STRATEGIES**

## *MEDLINE (1966 – November Week 2 2010); EMBASE (1980 – 20 10 Week 45); Medline In Process 17^th^ November 2010)*

**Ovid Multifile Search URL:** [**https://shibboleth.ovid.com/**](https://shibboleth.ovid.com/)

1 mcid.tw.

2 (target$ adj1 difference?).tw.

3 change score.tw.

4 change point.tw.

5 (clinical$ importan$ adj2 (difference? or change? or improvement? or effect?)).tw

6 (minim$ importan$ adj2 (difference? or change? or improvement? or effect?)).tw.

7 (clinical$ meaningful$ adj2 (difference? or change? or improvement? or effect?)).tw.

8 (minim$ meaningful$ adj2 (difference? or change? or improvement? or effect?)).tw.

9 (smallest meaningful$ adj2 (difference? or change? or improvement? or effect?)).tw.

10 (minim$ significant$ adj2 (difference? or change? or improvement? or effect?)).tw.

11 (smallest significant$ adj2 (difference? or change? or improvement? or effect?)).tw.

12 (minim$ detect$ adj2 (difference? or change? or improvement? or effect?)).tw.

13 (smallest detect$ adj2 (difference? or change? or improvement? or effect?)).tw.

14 (sufficient$ importan$ adj2 (difference? or change? or improvement? or effect?)).tw.

15 (sufficient$ meaningful$ adj2 (difference? or change? or improvement? or effect?)).tw.

16 (minim$ clinical$ adj2 (important or detectable or meaningful)).tw.

17 ((calculat$ or determin$ or comput$) adj2 meaningful).tw.

18 ((calculat$ or determin$ or comput$) adj2 detectable).tw.

19 ((calculat$ or determin$ or comput$) adj2 important adj2 (difference? or change? or improvement? or effect?)).tw.

20 ((calculat$ or determin$ or comput$) adj2 meaningful adj2 (difference? or change? or improvement? or effect?)).tw.

21 ((calculat$ or determin$ or comput$) adj2 detectable adj2 (difference? or change? or improvement? or effect?)).tw.

22 (definition$ adj2 (difference? or change? or improvement?)).tw.

25 ((responsiveness adj2 (calculat$ or determine$ or comput$)) and (measure$ or scale$ or score$ or rating$)).tw.

24 *sample size/

25 or/1-24

***PsycINFO (1967 to January Wk 2 2011)***

**Ovid Search URL:** [**https://shibboleth.ovid.com/**](https://shibboleth.ovid.com/)

1 mcid.tw.

2 (target$ adj1 difference?).tw.

3 change score.tw.

4 change point.tw

5 (clinical$ importan$ adj2 (difference? or change? or improvement? or effect?)).tw.

6 (minim$ importan$ adj2 (difference? or change? or improvement? or effect?)).tw.

7 (clinical$ meaningful$ adj2 (difference? or change? or improvement? or effect?)).tw.

8 (minim$ meaningful$ adj2 (difference? or change? or improvement? or effect?)).tw.

9 (smallest meaningful$ adj2 (difference? or change? or improvement? or effect?)).tw.

10 (minim$ significant$ adj2 (difference? or change? or improvement? or effect?)).tw.

11 (smallest significant$ adj2 (difference? or change? or improvement? or effect?)).tw.

12 (minim$ detect$ adj2 (difference? or change? or improvement? or effect?)).tw.

13 (sufficient$ importan$ adj2 (difference? or change? or improvement? or effect?)).tw.

14 (sufficient$ meaningful$ adj2 (difference? or change? or improvement? or effect?)).tw.

15 (minim$ clinical$ adj2 (important or detectable or meaningful)).tw.

16 ((calculat$ or determin$ or comput$) adj2 meaningful).tw.

17 ((calculat$ or determin$ or comput$) adj2 detectable).tw.

18 (smallest detect$ adj2 (difference? or change? or improvement? or effect?)).tw

19 ((calculat$ or determin$ or comput$) adj2 important adj2 (difference? or change? or improvement? or effect?)).tw.

20 ((calculat$ or determin$ or comput$) adj2 meaningful adj2 (difference? or change? or improvement? or effect?)).tw.

21 ((calculat$ or determin$ or comput$) adj2 detectable adj2 (difference? or change? or improvement? or effect?)).tw

22 *sample size/

23 (definition$ adj2 (difference? or change? or improvement?)).tw.

24 ((responsiveness adj2 (calculat$ or determine$ or comput$)) and (measure$ or scale$ or score$ or rating$ or metric$)).tw.

25 clinical$ importan$.id.

26 clinical$ significan$.id.

27 clinical$ meaningful$.id.

28 minim$ importan$.id.

29 minim$ significan$.id.

30 minim$ meaningful$.id.

31 smallest meaningful$.id.

32 smallest significan$.id

33 smallest importan$.id

34 sufficient$ importan$.id.

35 sufficient$ meaningful$.id.

36 minim$ clinical$.id

37 minim$ detect$.id.

38 importan$ difference$.id.

39 meaningful$ difference$.id.

40 minim$ difference$.id.

41 or/1-40

## *Cochrane Library (CENTRAL and Cochrane Methodology Register [CMR] Issue 1 2011)*

**URL:** [**http://www3.interscience.wiley.com/**](http://www3.interscience.wiley.com/)

#1 (mcid) or (target* NEXT difference*) or (change NEXT score) or (Change NEXT point)

#2 (clinical* NEXT importan*) NEAR/2 (difference* or change* or improvement* or effect*)

#3 (minim* NEXT importan*) NEAR/2 (difference* or change* or improvement* or effect*)

#4 (clinical* NEXT meaningful*) NEAR/2 (difference* or change* or improvement* or effect*)

#5 (minim* NEXT meaningful*) NEAR/2 (difference* or change* or improvement* or effect*)

#6 (smallest NEXT meaningful*) NEAR/2 (difference* or change* or improvement* or effect*)

#7 (minim* NEXT significant*) NEAR/2 (difference* or change* or improvement* or effect*)

#8 (smallest NEXT significant*) NEAR/2 (difference* or change* or improvement* or effect*)

#9 (minim* NEXT detect*) NEAR/2 (difference* or change* or improvement* or effect*)

#10 (smallest NEXT detect*) NEAR/2 (difference* or change* or improvement* or effect*)

#11 (sufficent* NEXT importan*) NEAR/2 (difference* or change* or improvement* or effect*)

#12 (sufficent* NEXT meaningful*) NEAR/2 (difference* or change* or improvement* or effect*)

#13 (minim* NEXT clinical*) NEAR/2 (difference* or change* or improvement* or effect*)

#14 (calculat* or determin* or comput*) NEAR/2 (meaningful)

#15 (calculat* or determin* or comput*) NEAR/2 (detectable)

#16 (calculat* or determin* or comput*) NEAR/2 (important)

#17 (calculat* or determin* or comput*) NEAR/2 (detectable)

#18 (definition*) NEAR/2 (difference* or change* or improvement*)

#19 (responsiveness) NEAR/2 (calculat* or determine* or comput*)

#20 MeSH descriptor Sample Size,

#21 (#1 OR #2 OR #3 OR #4 OR #5 OR #6 OR #7 OR #8 OR #9 OR #10 OR #11 OR #12 OR #13 OR #14 OR #15 OR #16 OR #17 OR #18 OR #19 OR #20)

## *Science Citation Index [SCI] (1970 - 22^nd^ January 2011)*

**ISI Web of Knowledge URL:** [**http://wok.mimas.ac.uk/**](http://wok.mimas.ac.uk/)

# 1 TS=mcid

# 2 TS="targeted difference*"

# 3 TS="change score"

# 4 TS="change score"

# 5 TS="change point"

# 6 TS="clinical* important" SAME TS=(change* or difference* or improvement* or effect*)

# 7 TS="minimal* important*" SAME TS=(change* or difference* or improvement* or effect

# 8 TS="clinical* meaningful" SAME TS=(change* or difference* or improvement* or effect*)

# 9 TS="minimal* meaningful" SAME TS=(change* or difference* or improvement* or effect*)

# 10 TS="smallest meaningful" SAME TS=(change* or difference* or improvement* or effect*)

# 11 TS="minimal* significant*" SAME TS=(change* or difference* or improvement* or effect*)

# 12 TS="smallest significant*" SAME TS=(change* or difference* or improvement* or effect*)

# 13 TS="minimal* detect*" SAME TS=(change* or difference* or improvement* or effect*)

# 14 TS="smallest detect*" SAME TS=(change* or difference* or improvement* or effect*)

# 15 TS="sufficient* important*" SAME TS=(change* or difference* or improvement* or effect*)

# 16 TS="sufficient* meaningful*" SAME TS=(change* or difference* or improvement* or effect*)

# 17 TS="minim* clinical* important"

# 18 TS="minim* clinical* detectable"

# 19 TS="minim* clinical* meaningful"

20 #1 or #2 or #3 or #4 or #5 or #6 or #7 or #8 or #9 or #10 or #11 or #12 or #13 or #14 or #15 or #16 or #17 or #18 or #19 AND Document Type=(Article)

***EconLit ( 1984- 31^st^ January 2011)***

**CSA Illumina URL: http://www.csa1.co.uk/**

S1 TX mcid or TX target* w2 difference*

S2 TX "change score" or TX "change point"

S3 TX minim* w2 importan or TX sufficient* w2 importan* or TX smallest w2 importan*

S4 TX minim* w2 meaningful* or TX smallest w2 meaningful* or TX sufficient* w2 meaningful*

S5 TX minim* w2 significant* or TX smallest w2 significant* or TX smallest w2 detect*

S6 TX minim* w2 detect* or TX minim* w2 difference or TX meaningful w2 difference

S7 TX minim* w2 change or TX smallest w2 difference -

S8 TX meaningful w2 change

S9 TX smallest w2 change

S10 S1 or S2 or S3 or S4 or S5 or S6 or S7 or S8 or S9

***ERIC (1960 – 28^th^ January 2011)***

**Proquest URL:** [**http://search.proquest.com/**](http://search.proquest.com/)

((mcid or (target* within 2 difference*)) or (("change score") or ("change point"))) or(minim* within 2 importan*) or(sufficient* within 2 importan*) or(smallest within 2 importan*) or(minim* within 2 meaningful*) or(smallest within 2 meaningful*) or(sufficient* within 2 meaningful*) or(minim* within 2 significant*) or(smallest within 2 significant*) or(smallest within 2 detect*) or(minim* within 2 detect*) or(minim* within 2 difference) or(minim* within 2 difference) or(meaningful within 2 difference) or(meaningful within 2 change) or(smallest within 2 change) or(important within 2 change) or(minim* within 2 change) or(smallest within 2 difference)

**Scopus (28^th^ Jan 2011)**

**URL:** [**http://www.scopus.com/**](http://www.scopus.com/)

((TITLE-ABS-KEY(mcid) AND DOCTYPE(ip)) or (TITLE-ABS-KEY("target* difference*") AND DOCTYPE(ip)) or (TITLE-ABS-KEY("change point") AND DOCTYPE(ip)) or (TITLE-ABS-KEY("minim* important") AND DOCTYPE(ip)) or (TITLE-ABS-KEY("minim* meaningful") AND DOCTYPE(ip)) or (TITLE-ABS-KEY("minim* significant") AND DOCTYPE(ip)) or (TITLE-ABS-KEY("minim* clinical*") AND DOCTYPE(ip)) or (TITLE-ABS-KEY("clinical* meaningful*") AND DOCTYPE(ip)))

***Clinical trial books/guidelines consulted***

Berry SM, Carlin BP, Lee JJ, Muller P. Bayesian adaptive methods for clinical trials. London : Taylor & Francis, 2011.

Chin RY. Principles and practice of clinical trial medicine. Amsterdam ; Boston: Elsevier, 2008.

Cleophas TJ, Zwinderman AH, Cleophas, TF, Cleophas EP. Statistics applied to clinical trials. London : Springer, 2009.

Cook TD, DeMets DL. Introduction to statistical methods for clinical trials. London : Chapman & Hall, 2008.

Friedman LM Furberg CD DeMets DL. Fundamentals of clinical trials. New York : Springer, 2010.

Hackshaw AK. A concise guide to clinical trials. Oxford:Wiley-Blackwell, 2009.

Julious SA. Designing clinical trials with uncertain estimates. London : University of London, 2006.

Machin D, Day S, GreenS (eds). Textbook of clinical trials. Chichester : John Wiley, 2006.

Matthews J. Introduction to randomized controlled trials. London : Chapman & Hall, 2006.

Peace KE, Chen D, Clinical trial methodology. London : Chapman & Hall, 2011.

Piantadosi S. Clinical trials: a methodologic perspective. Hoboken NJ: Wiley, 2005.

Pocock SJ. Clinical trials : a practical approach. Chichester : Wiley, 1983.

Spiegelhalter DJ, Abrams KR, Myles JP. Bayesian approaches to clinical trials and health-care evaluation. Hoboken NJ: Wiley, 2003.

Walters SJ. Quality of life outcomes in clinical trials and health-care evaluation: A practical guide to analysis and interpretation. Chichester: Wiley, 2009.

Wang D, Bakhai A. Clinical trials: a practical guide to design, analysis, and reporting. London: Remedica, 2006.

Statistical principles for clinical trials (E9): ICH tripartite guidelines [ document on the Internet]. Geneva: International Conference on Harmonisation of Technical Requirements for Registration of Pharmaceuticals for Human Use (ICH); 1998 [accessed March 2012]. URL: <http://www.ich.org/products/guidelines/efficacy/article/efficacy-guidelines.html>
